# Latent organizing for responding to emergencies: foundations for research

**DOI:** 10.1186/s41469-020-00074-z

**Published:** 2020-06-10

**Authors:** Paul C. van Fenema, A. Georges L. Romme

**Affiliations:** 1 Faculty of Military Sciences, Netherlands Defence Academy, P.O. Box 90004, 3509 AA Breda, The Netherlands; 2 School of Industrial Engineering, Eindhoven University of Technology, P.O. Box 513, 5600 MB Eindhoven, The Netherlands

**Keywords:** Organization design, Latent organizing, Resource management, Capability, Routines, Emergency response, Catastrophes

## Abstract

Time and again, many organizations and their staff members must respond to unexpected catastrophes like hurricanes (e.g., Katrina), virus pandemics (e.g., COVID-19), or other major emergencies. As a result, some organizations allow their employees to respond to external emergencies by engaging in response actions for a limited time, like in the case of emergency response teams. The latter teams consist of employees that act as emergency response officers who can respond to floods, train crashes, or other emergencies. Emergency response teams constitute an example of so-called latent organizing (LO) in the preparation for and response to any (unpredictable) future emergency. While latent organizing is ubiquitous in a societal and professional sense, it has hardly been studied in the organization design literature. In this paper, we develop a research agenda for studying LO. LO serves to prepare for and respond to emergencies, but otherwise remains largely dormant and inactive. When it is inactive, host organizations use the LO’s human and other resources for their own gainful purposes. Resources for LO are thus organized in a quasi-permanent fashion, one that is rather latent until activated by an emergency. We further develop the construct of latency to explore how effective LO can be designed and facilitated. In addition, we develop a research agenda for future work in this area.

## Introduction

Across the globe, many organizations and their staff members have recently been acting and improvising in response to the COVID-19 pandemic. The lack of disinfection fluids motivated several beer brewers to (temporarily) reconfigure their production process to produce hand sanitizer gel. In the Netherlands, units of the Dutch army were assigned to coordinate and facilitate the transport of COVID-infected patients between hospitals.^1^ These are examples of latent organizing in response to unpredictable emergencies (van Aken and van Fenema 2014). Well-known examples of latent organizing are emergency situations in which people employed by other (so-called host) organizations quickly come together to act as emergency response teams on the sites of major train crashes, floods, or other disasters (Salmon et al. 2011).

The unpredictability of the responses to this type of emergency situations poses fundamentally new challenges. One key challenge is to keep the activation delay of the emergency response as short as possible (Schakel et al. 2016). Very short activation delays may make emergency response teams more effective, but typically also raise prohibitive costs. For example, these prohibitive costs arise when members of emergency response teams use work time and organizational resources to learn various action skills and coordination procedures, which are relevant in emergency situations but have no economic value for the host organization (employer).

Whereas there has been some work on latency in organization studies (Ebbers and Wijnberg 2009; Starkey et al. 2000; Westphal et al. 2001), the notion of latent organizing is underdeveloped, especially when switching to another task domain is required. In this article, we therefore explore deliberately designed *latent organizing* in the context of preparing for and responding to emergency situations. We will first discuss the notion of latency in general and in the domain of organizations, and then define the nature of deliberately designed versus emerging forms of latent organizing. Subsequently, we explore the strategy of latent organizing and the various reasons to use this strategy; we also identify key issues in creating effective latent organizing. Finally, we use these insights to develop a research agenda for developing robust theories of this novel type of organization as well as design principles for establishing and sustaining effective forms of latent organizing.

## Latent organizing

Organizing in response to specific demands is a common strategy in industry: instead of the common make-to-stock strategy, a company can adopt a so-called make-to-order production strategy, if its assortment flexibility is low (Priem and Swink 2007). Another widespread form of organizing for action-on-demand is the matrix structure adopted in many product development (departments in) firms: for each new product design “order” received, a project team is composed from members of various disciplines, depending on the nature of the assignment (Ford and Randolph 1992; Joyce 1986). In both examples, most resources such as competent people and dedicated equipment are permanently available, which enables immediate action when an order arrives. Moreover, in both cases the demand is rather easy to predict, and peaks in demand can be handled by processing orders sequentially and/or by adding (staff) capacity.

Many organizations also draw on temporary organizing in projects (Bakker et al. 2016; Kenis et al. 2009). These dedicated project teams or taskforces are often set up to address strategic challenges by developing solutions, such as new service designs or organizational change interventions (Lawson et al. 2009; Vlaar et al. 2008). Typically, these project teams are formed by selecting various staff members that, for a limited time, work on the project full- or part-time.

However, there are urgent situations in which (people employed by) organizations need to respond immediately. For example, emergency response teams can act quickly to provide first-aid and other services at the sites of highly unpredictable emergencies like train crashes or floods (Salmon et al. 2011). The high unpredictability and (relative) low frequency of these emergencies pose fundamentally new challenges. The costs arising from permanent resources fully dedicated to emergency response units are prohibitive, whereas the ad hoc acquisition and organization of the required resources (in response to an immediate emergency) tends to prove rather ineffective.

Other examples of latent organizing include emergency response units such as (voluntary) fire brigades and riot police. In case of the riot police, it is apparent that having a permanent unit for dealing with riots (within the police force) is far too expensive: riots are very unpredictable, as are the exact nature, location, and magnitude of any riot. But, once a major riot breaks out and an immediate demand for police action arises, the activation delay of this action or switching effort needs to be as short as possible (Schakel et al. 2016). That is, a sufficient number of police officers need to be assembled from local, adjacent, or distant police forces and mobilized as soon as possible. The options for this challenge can be positioned on a spectrum, ranging from establishing and using completely dedicated resources for riot management to no dedicated resources at all. At the dedicated extreme, one can establish a specialized riot police department, which has the advantages of a short activation delay, specially trained personnel, optimal procedures for communication and coordination, and specialized equipment and transportation vehicles. The main disadvantage is the (typically) prohibitive costs of these resources arising from long periods of inactivity. At the ad hoc extreme, riot teams are assembled from any available pool of police officers. This is a more cost-effective solution but implies longer activation delays and lower effectiveness in comparison to well-trained dedicated riot units.

The middle way between these two extremes is to prepare for responding to (external) emergencies in the form of a *latent organizing* (*LO*). Police officers are assigned to a riot unit and trained in various action, communication, and coordination procedures, while performing their regular duties during the inactive state of the riot unit. Compared to a completely dedicated unit, the latent unit is much more cost-effective. Compared to the ad hoc unit, the latent unit has a shorter activation delay and is likely to be better prepared and thus more effective in handling riots or other emergencies. Of course, the choice between these three options also depends on the expectations with respect to the frequency and magnitude of any emergency.

### Latency in organization studies

A latent object or process is one that exists but is not (yet) manifest. Latency cannot be equated with “potentiality” or affordance (Leonardi 2017): a latent object exists, but is not manifest, while potential and affordance refer to something not yet existing but possibly coming into existence. Thus, latency is a silent process/object that can be activated as a novel reality, while potentiality and affordance merely refer to becoming (Bjerregaard and Jonasson 2014; Leonardi 2017). In engineering, latency refers to the time between a cause and an effect in physical systems, for example, the time between the sending of a signal and its reception: here, the cause (i.e., sending) immediately triggers a process in the system, but the effect is not yet manifest for the receiver (Blake 2003). In biology and medicine, latency refers to incubation time; for example, the delay between an infection and the first observed symptoms, or the time between taking a particular medicine and perceiving its effects (Chan and Johansson 2012). Latency in the field of information systems refers to the time one needs to retrieve requisite data. A zero-latency enterprise is one in which a business event, recorded somewhere in the enterprise, triggers appropriate actions across the entire enterprise without any substantial delay (Langlois and Chauvel 2017; Nguyen et al. 2007). Thus, backup systems, used by hospitals for quick response to power failures, can be regarded latent systems, while they may have the potential and affordance of use beyond a primary devised function. A latent phenomenon is intermittently manifested in relation to ongoing processes and use of resources: for example, some highways are designed to be used as airstrips-on-demand for (military) aircraft; the highway then provides (and is prepared and maintained as) a latent airstrip, which only becomes manifest on demand and at all other times is used by cars and trucks.

Organizational latency can refer to any existing but not manifest roles, routines, services, organizations, and inter-organizational networks (Becker et al. 2013; van Fenema et al. 2014). While organizational latency is a ubiquitous phenomenon, there is hardly any literature about it. Starkey et al. (2000) were the first to use the term “latent” in the context of organizations. They discussed latent networks in the British TV world. Inspired by Starkey and co-authors Ebbers and Wijnberg (2009) discussed similar networks in the Dutch TV world (Ebbers and Wijnberg 2009). In these two articles, latent networks involve informal networks of social relations emerging among individuals. These latent social networks emerge from cooperation in one or more projects but are sustained beyond these projects. Hence, these networks can be activated when at a later stage a demand for them arises.

Similarly, latent network ties have been defined as ties that were previously created but are currently inactive (Mariotti and Delbridge 2012). Usually, these networks—called ephemeral ones (Lanzara 1983)—partially dissolve after a period of active use, such as a project. In this article, we do not address emergent, informal, or ephemeral social structures, but focus on the *deliberate* design of permanent latent organizing in the context of organizing for unpredictable action. Moreover, we assume a switch of task domains.

### Latent organizing defined

**Fig. 1:**
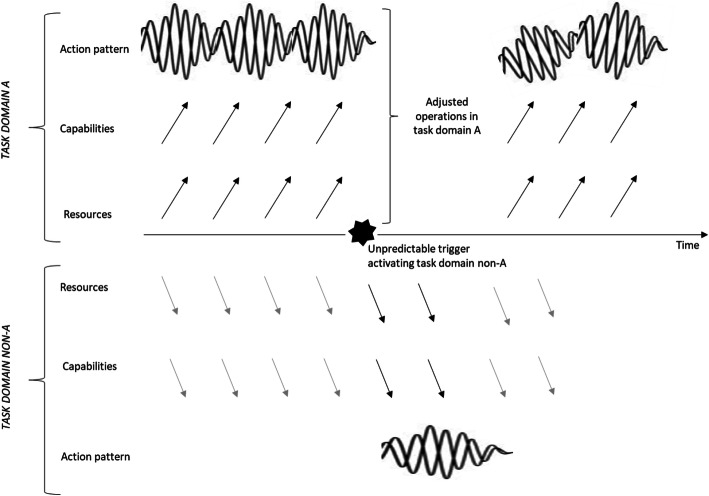
Latent organizing defined

An example of latent organizing is the response of various enterprises to emergency situations (Rodriguez et al. 2006), such as Katrina and the current COVID-19 crisis. In these emergency situations, some enterprises take on different roles for the common good, in addition to adapting their normal business activities. For example, in the recent COVID crisis, British supermarkets were urged and struggled to adapt their home delivery services (beyond their regular home delivery routine) to prioritize vulnerable people in home quarantine and NHS staff.^2^ Another example when the COVID pandemic in March 2020 broke lose is the major changes that hotels had to make in their services and facilities; they started accommodating many guests with symptoms of the coronavirus who got stuck in their hotel rooms, or even adjusted their operation to a hospital-like overflow care setting for patients. These enterprises thus must activate alternative configurations of actors and resources within their organization, to generate temporary operations that fundamentally differ from their usual business. Figure 1 outlines the nature of latent organizing as consisting of resources, capabilities, and action patterns in the host company’s normal task *domain A* versus temporary activation of some of these resources and capabilities in a rather unpredictable task *domain non-A*. Accordingly, we define *latent organizing* as involving resources and capabilities that remain dormant in the context of the regular task domain A of host organizations, until they are activated by an emergency or crisis situation (the non-A domain) that cannot be addressed within task domain A. The resources and capabilities include (people with) professional skills, routines, organizational networks, and other resources and capabilities (Becker et al. 2013; van Fenema et al. 2014).

Our definition of LO encompasses three specific elements that serve to capture the latency phenomenon and demarcate its boundaries. First, while latent organizing is *not active in the context of the host organization*’*s A domain*, *it is manifested upon activation in the non-A domain*. For instance, some hotels during Katrina shifted to a new policy of accepting only stranded guests and proving “help of different kinds for employees’ loss of property and personal possessions” (Rodriguez et al. 2006: 89). As Fig. 1 illustrates, host organizations normally act in their task domain A (above the *X*-axis) and then hardly spend efforts on non-A (below the *X*-axis), although some level of preparedness needs to exist. If organizations have no preparation whatsoever for non-A, they can only improvise in response to emergency situations—which is not what we would call latent organizing.

Second, LO serves to *switch from the common task domain A towards the non-A domain*. Consequently, latent organizing is not about switching between regular tasks in domain A. For example, in 2020 many hospitals in Europe were taken by surprise by the large amounts of COVID-infected patients brought into their intensive care (IC) units, forcing them to increase IC capacity and shift staff and other resources from other units to the IC. This is an example of major shifts *within* the regular task domain A of a hospital (Schakel et al. 2016). Other examples that have also been observed during the corona crisis include collaboration among partner organizations (Horwitz and McGahan 2019) and backing-up or scaling up existing organizational activities (Huang et al. 2017). Our definition of latent organizing also excludes activation of a host organization’s capability in its regular task domain, such as the “deployment” of a military unit (Biscop 2015), crisis response within the regular task domain (Kranz 2001), or developing a novel proposition (e.g., TV show) that fits within the boundaries of that domain (Ebbers and Wijnberg 2009). Moreover, our definition of LO also excludes temporary interruptions of organizational activities due to seasonal fluctuations, festivities, or maintenance work.

Third and finally, LO is activated in the non-A domain by any *unpredictable emergency or crisis that cannot be addressed within domain A*. What is demanded of, for example, emergency response teams is unknown in advance. This only becomes (somewhat) clear when they arrive at the spot, for instance the train crash or the flooded neighborhood. This activation mechanism almost by definition comes with a high sense of urgency and has an unknown (though limited) duration. Eventually, the people involved will return to their normal operations (in task domain A), such as hotel service staff re-assuming their tasks in providing commercial spaces for paying guests (Rodriguez et al. 2006). The emergency/crisis element in our definition thus excludes longer-term change processes, such as the gradual shift of an enterprise towards a new (type of) business model (Chesbrough et al. 2013; Pache and Santos 2013).

The above definition of LO also serves to demarcate it from permanent organizational routines, consisting of performative and ostensive aspects that influence each over time (Feldman 2000). These routines are continually “recreated,” as the organization interprets contextual cues (Dionysiou and Tsoukas 2013) and operates in a given institutional setting (Smets et al. 2017). Organizational routines are thus mostly enacted by dedicated resources allocated to perform within task domain A. By contrast, latent forms of organizing lack the continual performance and ostensive evolution in a regular task domain, because they are only activated in exceptional and unpredictable emergency situations. For example, those volunteering for emergency response teams may have some access to basic training and exercises, but true routine development is impossible because their enactment is continually broken in pieces. The latter “brokenness” may significantly differ between emergency response teams in different industries and/or countries: some may hardly be exposed to training and exercises in how to apply their resources and capabilities, whereas other teams cultivate their (emerging) routines by training and performing exercises—for example, by developing and testing new procedures for how to handle large fires (Weick 1993). In general, compared to permanent organizational routines in the regular domain A, latent organizing suffers from routine brokenness because its capabilities and resources can only be incidentally applied to emergencies in the non-A domain (see Fig. 1).

Moreover, LO also differs from purely improvisational organizing in task domain non-A. The extant literature on emergency response tends to mainly address improvisational organizing in teams (Meyerson et al. 1996) and across organizational boundaries (Ansell et al. 2010). As argued earlier, LO is typically established when, on one hand, an emergency response requires resources that are too expensive to remain idle during the latent state because of their magnitude or specificity, while on the other hand, any activation of these resources needs to be swift and (almost) immediately effective. For instance, most fire brigades in cities involve full-time employed professionals, while in the countryside one tends to use latent organizing in the form of volunteer fire brigades that are only activated in case of an emergency. In the countryside, fires are too infrequent to justify a fully dedicated firefighting organization, which implies volunteers need to be trained to enable their (almost) immediately effective deployment in response to fires or other emergencies.

### Activation

**Fig. 2:**
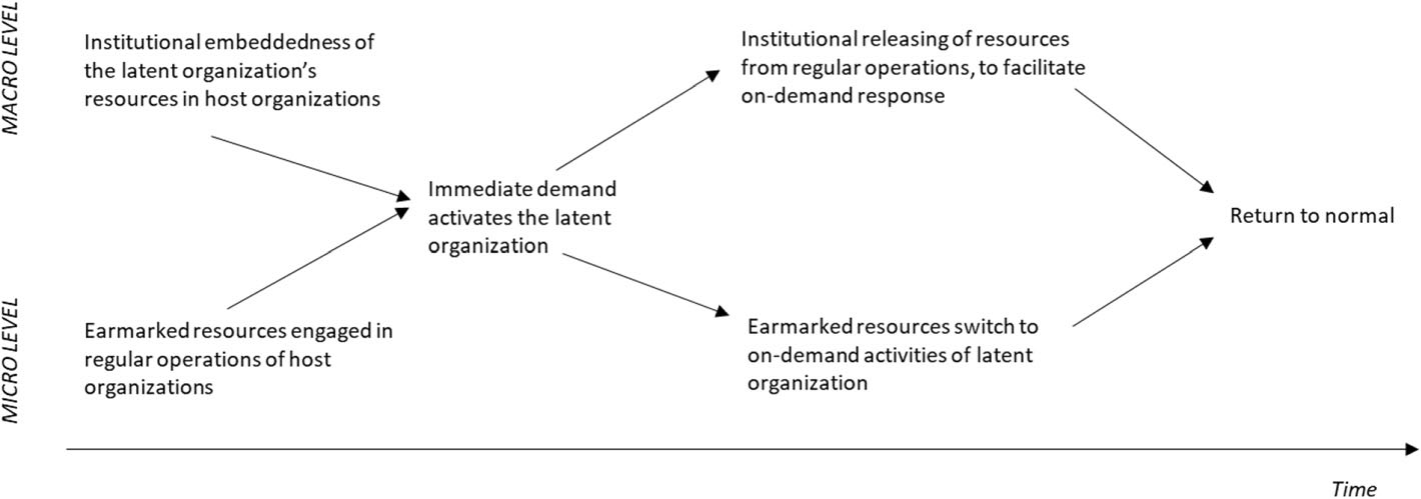
The activation process of latent organizing

LO is established to limit the time needed for acquiring and mobilizing the required resources. It thus needs to have an adequate management system in place (almost) immediately upon activation. At that moment, the unit (e.g., fire brigade) becomes quickly effective and generates new experiences as a platform for learning and further professionalization. In the inactive state of LO, its main resources are embedded in one or more designated host organizations or distributed across undesignated host organizations, like in the case of volunteer fire brigades or military reserves (Edmunds, Dawes, Higate, Jenkings, & Woodward, 2016). In the latter case, the resources and capabilities are earmarked to be used by the LO (following institutional agreements or legal requirements), once an immediate need for action emerges. When the LO is inactive, the host organizations use these human resources for their own purposes. At the micro level of individual staff members, human resources thus move between the host organization and the (activated) latent organization. Figure 2 visualizes these dynamics over time, at both the institutional macro-level and micro-level of actions (Smets and Jarzabkowski 2013).

### Process of reification in latent organizing

Several examples serve to demonstrate that LO may differ in terms of the level of reification.^3^ For one, SWAT teams are often composed of not fully dedicated people. These SWAT team members consist of law-enforcement professionals that have regular jobs within various departments of the police force, but are trained for SWAT assignments (Bechky and Okhuysen 2011). Police officers with SWAT qualifications are likely to consider these arrangements as another (but irregular) element of their roles. As such, the realness of latent organizing for SWAT is never questioned and is internalized by all actors to the extent that they can respond rather effectively, with a short activation delay. These differences in awareness and commitment can be interpreted as differences in how participants and other stakeholders *reify* (or “make into a thing”) these LO arrangements for emergency response.

Another example is the response by supermarkets in the recent COVID-19 pandemic. In this pandemic, governmental stakeholders and the public at large expected new services from supermarkets and other grocery stores in the area of vital supplies and public health.^4^ The level of reification of LO responses to a pandemic by grocery stores is lower than in the case of SWAT teams, due to the infrequent nature of this type of crisis, and legal restrictions in charging commercial enterprises with public-collective responsibilities (Herzig and Moon 2013; Waddock 2008).

We interpret reification as a social process of transforming interpersonal relationships into a form of organization that has instrumental and autonomous qualities (Chang 1985). In contrast to an ad hoc assembled team, an individual’s own labor becomes something objective and independent of him/her in LO. This objective thing then controls the individual, by virtue of the LO becoming an autonomous entity (Lukács 2017). So, LO avant la lettre can be positioned on a continuum from a relatively weak reification (i.e., relying merely on trust and professional identity {Majchrzak, 2007 #4233}) to a rather strong one (i.e., professionals earmarked for LO’s emergency responses).

Reification is a common and usually spontaneous process (Lane et al. 2006), driven by continual communication and cooperation between insiders and enhanced by interactions with outsiders, who also regard the organization as an “objective” entity with a particular status, history, and creditworthiness (Silverman 1970). Silverman (1970) considered the reification of organizations as a bad phenomenon, because it may impede freedom of individual action as well as organizational change and innovation. However, LO is exposed to huge risks if its emergency response routines are not fully embraced. These routines may erode, especially during long periods of inaction (Bigley and Roberts 2001; Boin and 't Hart 2009). Deliberately designed forms of reification may prevent this erosion by enabling both insiders and outsiders to identify with the latent organization and develop its capabilities for emergency response.

### Example: latent organizing in emergency preparation

The emergency response to hurricane Katrina illustrates the reasons for establishing latent organizing for emergency preparation. Katrina, a hurricane of category 3 with wind speeds of up to 200 km/h, combined with storm surges of up to 5 m, passed southeast of New Orleans in August 2005.^5^ More than 20 levees and floodwalls were breached, and more than 80% of New Orleans was flooded. Almost 90% of all New Orleans residents were evacuated before Katrina arrived, but it still caused more than 1400 fatalities and a damage of around 70 billion USD (WhiteHouse 2006).^6^

Afterwards, the Katrina emergency response was heavily criticized (Congress 2006; GAO 2006): response agencies were said to lack proper qualifications and training, and the resources of the Federal Emergency Management Agency were not adequately deployed. In terms of the institutional context (i.e., macro-level) in Fig. 2, the Department of Defense had to wait for written requests from the Department of Homeland Security before it could engage in response actions, also because of strict legal regulations regarding military deployment on national soil (van Fenema 2012). Moreover, arrangements for cooperation between federal, state, and local agencies existed on paper, but did not work well in practice and were marred by jurisdiction issues. Overall, the emergency preparation regarding Katrina involved an elaborate system of agreements, contracts, and laws, whereas the actual emergency response involved a largely ad hoc approach.

The Katrina disaster did trigger significant improvements. Many of these improvements have been about organizing formal communication and control systems, like the National Response Plan, the National Incident Management System, and the Incident Command System.^7^ Our argument thus far suggests that the design of such formal systems and arrangements, while important, is not enough for LO to effectively come alive. These formal arrangements need to be complemented with a more comprehensive design perspective. This would undergird latent organizational capabilities and draw on widely distributed resources (of many, also local, host organizations), thereby enabling a more powerful response to large-scale emergencies. In this respect, LO can be regarded as a complementary solution for responding to disasters like Katrina or the 2020 COVID-19 pandemic.

If LO practices are fully developed, the professionals involved are committed to these practices. That is, they have been trained in action, and managerial and communication procedures, resulting in a sound internalization thereof. This enables these professionals to not just follow procedures, but also to act on their own initiative and improvise whenever the need to do so arises (Weick et al. 1999). Furthermore, LO can be developed to be “relatable” in the sense of being able to work with others. Here, members of emergency response teams and other forms of LO need to be competent in setting up ad hoc collaboration with non-professional actors, whose roles and behaviors—as the Katrina case shows (Congress 2006)—are difficult to predict and include in any emergency response system.

## Towards a research agenda

To develop a research agenda, we explore key theoretical issues in designing and facilitating LO in this section.

### Resource management processes and latent organizing

LO can be regarded as a specific instantiation of resource management. Many of the design challenges regarding any complex organization also arise in designing for LO. But the unique characteristics of LO give rise to several specific issues in the area of resource management.

In this respect, the resource-based view (RBV) appears to offer a relevant theoretical perspective (Kraaijenbrink et al. 2010; Lai et al. 2012; Silverman 2017) for understanding LO. After all, with LO, one typically faces major challenges in using and activating non-dedicated resources, like volunteer-members of fire brigades or emergency response teams. In RBV, a company is conceptualized as a bundle of heterogeneous resources (Nagano 2020). RBV researchers distinguish a number of resource management processes aimed to create and sustain competitive advantage (Sirmon et al. 2007). For LO, we assume that “competitive advantage” is not a primary orientation. In response to emergencies, LO professionals temporarily stop their work (for their employer, as host organization) in task domain A to help address emergency situations that, by definition, represent non-A challenges. With this strategic orientation in mind, we are interested in resource processes that undergird LO capabilities (Fig. 1). The following resource management processes can thus be distinguished.

- *Structuring* the resource portfolio—this refers to sourcing of, on the one hand, resources deemed essential for accomplishing the mission of the LO entity (e.g., first-aid equipment, transport vehicles), and on the other hand support resources needed for communication, coordination, and administration (Bower and Gilbert 2005; Strikwerda 2017: 48).
- *Bundling* resources—integrating the acquired resources and building capabilities, relevant for organizational effectiveness (Bower and Gilbert 2005; Sirmon et al. 2007). Effective bundling of resources involves the development of collective knowledge and skills through learning by doing. Here, bundling resources is more than sharing individual knowledge. Collective resources and capabilities for LO are emergent in nature and embedded in institutionalized practices (e.g., of host organizations).
- *Leveraging* resources—mobilizing, deploying, and coordinating the bundle of resources. Bundling and leveraging resources are a process that is similar to strategy execution in the resource allocation literature (Bower and Gilbert 2005).

In designing and developing LO, major challenges and issues arising from these three dimensions of RBV need to be addressed. First, regarding *acquisition*, the LO entity does not own the (vast majority of the) resources it uses, but host organizations do. The LO entity thus would need to acquire and contract these resources from the host organizations, which raises the question: who is going to act on behalf of the LO actors in making contracts with host organizations? One resource governance solution here could be that the LO entity obtains a separate legal basis (e.g., including all emergency response teams in region *X*). Another solution is that a governmental (e.g., municipal) agency would contract resources from host organizations, like in the case of a small municipality that sets up a fire brigade composed of volunteers employed by various companies in the same village. Without a separate entity acting on behalf of the LO, a less solid base appears to exist for responding to emergencies. Without such an agency, each company or other organization will seek to focus its emergency management capacity on the more predictable type of emergency. For example, hotels develop an internal capability for first-aid but will normally avoid investing in resources and capabilities needed to respond to major catastrophes outside the hotel’s premises (Rodriguez et al. 2006: 89). Notably, funding LO is often difficult, because host organizations and other stakeholders may not have a surplus of resources, and the economic incentive to contribute resources tends to be weak. In general, the sourcing and basic governance of resources appears to be a vulnerable dimension in creating and sustaining LO for emergency response.

Second, resource *bundling* in LO involves first and foremost the need to enhance the reification of its mission regarding emergency response (in non-A task domain), to make its members and external stakeholders recognize it as a real organization (e.g., a fire brigade composed of volunteers), be it one that may be inactive for long timespans. Another challenge here is how to integrate the resources made available by a (possibly very) large number of host organizations into a collective capability, and especially how to develop this capability while the LO is in an inactive state. For example, in the case of building LO for emergency response, training-on-the-job is typically not possible in the host organization and would be highly inefficient if it were possible. Learning-by-doing thus needs to be facilitated in other ways, for instance, by training people in virtual microworlds (cf. flight simulators) in which they are exposed to realistic scenarios and learn various response tactics and strategies by trying them out (Romme 2003). Hence, building up capabilities is a fragile process, echoing the routine brokenness earlier mentioned. Since the integration and capability development of resources must aim at a particular level of expected effectiveness, this raises the question as to how one can measure and predict the effectiveness of any (inactive) LO in its active state. We already mentioned that LO for emergency response is a public service that does not aim at profitability or competitive advantage. Moreover, participants in LO may remain responsible for activities in their regular task domain A, even when engaged in an emergency response. Think of a general medical practitioner who joins the emergency response team acting on the site of a major train crash, but who also remains available for (emergency) calls from his regular clients. Hence, key actors in LO are likely to be exposed to tensions between (or even a conflict of) the two masters they are trying to serve. In designing the LO capability, its expected level of effectiveness in terms of the activation delay is key: how soon (in minutes, hours, days), after an emergency arises, should a particular level and volume of emergency response action be reached? Moreover, as previously explored, future research also needs to explore how LO can reduce the risk of eroding reification and readiness for action in long times of inactivity.

With regard to *leveraging* resources, an LO entity may benefit more from access to well-trained people rather than a detailed system of formal contracts and agreements (Bigley and Roberts 2001), in order to make it highly responsive to unexpected emergencies. Responses to a wide range of nature-driven or human-made catastrophes cannot be prepared by detailed scenarios, meticulous planning, and elaborate control systems—which are all likely to remain paper tigers (Brattberg and Rhinard 2012). The capabilities of LO depend on the swift activation and on-the-spot performance of skilled people, rather than ongoing routines (Feldman 2000). Here, effective responses draw on the real-time judgement, local insights, and improvisational skills of a large group of trained people (Weick and Sutcliffe 2007). Consequently, the communication and coordination systems used for leveraging the resources of LO for emergency preparation should reflect this essential point. As mentioned earlier, LO can be positioned between permanent organizations delivering an ongoing performance based on the resources acquired and improvisational organizations predominantly relying on ad hoc collaboration and resource combination processes. Table 1 provides an overview of resource management challenges for LO.

**Table 1:** Resource management in latent organizing: key challenges

Resource management process	Key challenge for latent organizing
Acquisition	• The LO does not own the resources; resource acquisition is based on contracting the services from the owners of the resources (i.e., host organizations). • A key question is who acts on behalf of LO’s mission for emergency response, by acquiring key resources? A separate legal entity or a governmental agency? • Funding resources is difficult because the economic motive to contribute is typically weak.
Bundling	• A key aspect of resource bundling is enhancing the reification of the organizational mission of preparing for emergency response, to make its members and external stakeholders recognize it as a real organization (that may be inactive for long timespans). • Another challenge is how to integrate resources from a (possibly very) large number of host organizations, that is, how to develop the required capabilities while the organization is inactive, because training-on-the-job (in the host organizations) is typically not possible, learning-by-doing needs to be facilitated in other ways (e.g., in virtual microworlds). • The integration and capability development of resources needs to aim at an expected level of effectiveness. This raises questions regarding how to measure and predict the effectiveness of the active state of the organization when it is inactive, especially what is the activation delay (in minutes, hours, days) toward a particular level and volume of emergency response? • How does/can the organization reduce the risk of erosion in its reification and readiness in long times of inactivity?
Leveraging	• Responsiveness to unpredictable emergencies arises from (a) access to well-trained people, rather than a detailed system of formal contracts and agreements and (b) real-time judgement, local insights, and improvisational abilities of these people. • Consequently, the communication and coordination systems used for leveraging these human resources (e.g., for emergency preparation) should reflect this essential starting point.

### Hierarchies and platforms

Our argument was thus far largely informed by RBV, but other theories such as those in the area of hierarchy (e.g., Billinger and Workiewicz 2019), platforms (Luo et al. 2018), and resilience (e.g., Välikangas and Romme 2013) can provide complementary perspectives. In this respect, the societal need for LO for emergency response raises important other questions. For example, what type of hierarchy best enables the activation decision that turns an LO into its active state, and what type of minimal hierarchy is required at the level of groups of professionals responding to emergency situations (cf. Burton et al. 2017; Romme 2019). Moreover, how do hierarchical differences between people in the host organization (task domain A) affect their decisions and actions in a coordinated response to an emergency outside regular domain A (cf. Nobles 2019)?

Similarly, the literature about open platforms (Luo et al. 2018) can inform future work in exploring what latent and platform organizations have in common and where they diverge. While platforms may facilitate activities within a rather homogeneous task domain, such as retail, accommodation, mobility, or food delivery, their role in emergency situations warrants attention. Generic capabilities may prove useful in organizing scattered resources. Other interesting questions are how can semi-dedicated resources be organized as for instance a hybrid organization (Pache and Santos 2013); to what extent are latent organizing actors aware of their institutional embeddedness? And what could be the role of (dual) professional identity and swift trust in activating resources in a socially effective manner (Meyerson et al. 1996)?

### Learning

While actors engage in non-A activities, they are likely to learn. This type of learning differs from intra-task domain learning, such as fire brigades that learn about and improve the procedures for handling huge fires (Weick 1993). The literature on high reliability organizing advocates a “mindful infrastructure” that serves to manage failures, resist oversimplification, and remain sensitive to operational experiences (Weick and Sutcliffe 2007). Development of such an infrastructure can have major impact over time, also for the host organizations’ processes. Demarcations between the A and non-A domain may shift or become more ambiguous, as people start using their acquired experiences and skills after returning to their normal task domain A (Puranam et al. 2014). Moreover, action patterns in the A and non-A domains may overlap, in terms of a more generic set of resources and capabilities in areas like leadership, problem solving, goal setting, and teamwork. This generic set may, in turn, serve to create a more solid foundation for switching between regular work and emergency response action (Edwards and Bruce 2004). Learning from LO work may thus focus on this generic set of resources and capabilities as well its application in different task domains.

### Research methods for understanding and improving latent organizing

We would suggest two types of research methods to address the research challenges and questions described thus far. The first type of method would involve studying extant LO for responding to emergencies, aiming to *understand* the problems of establishing and developing such organizational forms as well as the measures taken to deal with these issues. This type of future work will draw on (comparative) in-depth case studies, surveys, and other methods to collect and analyze data. Notably, there are as hardly any organizations formally labelled as *latent*, but many self-organized initiatives and programs for responding to emergency situations appear to have the characteristics of LO as defined earlier in this paper.

The second type of method involves action research (James et al. 2011), design science (Romme and Reymen 2018), and similar approaches to improve (existing or future) designs, processes, and structures for creating and sustaining LO together with professionals in the field. As such, this type of research aims at further extending the notion of LO as well as developing blueprints that can inform future designs of LO. Action research may be useful (James et al. 2011) to enable researchers to work together with professionals in emergency preparation and other areas in which LO is critical. Researchers and practitioners can thus develop more effective practices in preparing and responding to emergency. Professionals may contribute domain and local knowledge to this emerging body-of-knowledge, while researchers can seek to further develop actionable knowledge on LO. Actionable knowledge on LO can also be developed by means of design science (Romme and Reymen 2018) and related methods to develop improved designs, processes, and structures for creating and sustaining LO—again in close collaboration with professionals in the field. For example, studies drawing on design science can serve to develop coherent sets of principles for redesigning current forms of LO or designing entirely new ones (Knight et al. 2020; Romme and Endenburg 2006; Romme 2016).

## Conclusion

While latent organizing for responding to emergencies is ubiquitous in a societal and professional sense, it has hardly been studied in the organization design literature. In this paper, we have therefore explored and discussed LO for responding to emergencies. LO can be largely informal in nature, but we focused on the deliberate design of latent systems with critical resources and capabilities for emergency management. Subsequently, we developed a research agenda for studying LO.

## Data Availability

Data sharing is not applicable to this conceptual article, as no datasets were generated or analyzed during the current study.

## Footnotes

1 https://nypost.com/2020/03/21/dutch-military-deployed-to-help-coronavirus-victims-in-the-netherlands/

2 https://www.bbc.com/news/uk-scotland-scotland-business-52122376; https://www.dailymail.co.uk/news/article-8167095/Thousands-pensioners-unable-online-supermarket-delivery-key-workers-turned-away.html

3 We use reification in a neutral sense as existence separate from initial authorship {Lane, 2006 #263}.

4 https://www.theguardian.com/business/2020/mar/06/uk-supermarkets-asked-to-deliver-food-to-people-in-coronavirus-self-isolation

5 https://www.nhc.noaa.gov/data/tcr/AL122005_Katrina.pdf

6 https://biotech.law.lsu.edu/katrina/ipet/Volume%20I%20FINAL%2023Jun09%20mh.pdf

7 https://www.hsdl.org/?view&did=735934
